# Supplementary material for: Impact of the Uncoupling Protein 1 on Cardiovascular Risk in Patients with Rheumatoid Arthritis
Source: Cells. 2021 May 7;10(5):1131. doi: 10.3390/cells10051131 (PMC8151725; doi:10.3390/cells10051131)
Supplement: Supplementary file 1 [file cells-10-01131-s001.zip › cells-1173054-supplementary.pdf]

cells

MDPI

Supplementary Material

Impact of the Uncoupling Protein 1 on Cardiovascular Risk in Patients with Rheumatoid Arthritis

Lovisa I. Lyngfelt [1,*], Malin C. Erlandsson [1,2], Mitra Nadali [1,2], Shahram Hedjazifar [3], Rille Pullerits [1,2,4], Karin M. Andersson [1], Petra Brembeck [3], Sofia Töyrä Silfverswärd [1], Ulf Smith [3] and Maria I. Bokarewa [1,2]

1 Department of Rheumatology and Inflammation Research, Institute of Medicine, University of Gothenburg, 405 30 Gothenburg, Sweden; malin.erlandsson@rheuma.gu.se (M.C.E.); mitra.nadali@vgregion.se (M.N.); rille.pullerits@rheuma.gu.se (R.P.); karin.andersson@rheuma.gu.se (K.M.A.); sofia.silfversward@rheuma.gu.se (S.T.S.); maria.bokarewa@rheuma.gu.se (M.I.B.)
2 Rheumatology Clinic, Sahlgrenska University Hospital, 413 45 Gothenburg, Sweden
3 Lundberg Laboratory for Diabetes Research, Department of Molecular and Clinical Medicine, Institute of Medicine, University of Gothenburg, 405 30 Gothenburg, Sweden; shahram.hedjazifar@astrazeneca.com (S.H.); petra.brembeck@gu.se (P.B.); ulf.smith@gu.se (U.S.)
4 Department of Clinical Immunology and Transfusion Medicine, Sahlgrenska University Hospital, 413 45 Gothenburg, Sweden
* Correspondence: lovisa.lyngfelt@gu.se; Tel.: +46737198781

Table S1. Primer pair sequences.

S1A Assay on pre primed plate from SA Biosciences (Qiagen).

	**Cat.nr. (SABioscience, Qiagen)**
RETN	PPH02309B
STAT3	PPH00708F
ACTB	PPH00073G
POLR2A	PPH13508E

S1B Individual primer sequences

**UCP1 F2**	**CTGGAATAGCGGCGTGCTT**
UCP1 R2	AATAACACTGGACGTCGGGC
ABCA1 F1	GAGTGAAGCCTGTCATCTACTGG
ABCA1 R1	GGAGGACACATAGGACTTCTGC
Leptin F1	TGTCACCAGGATCAATGACA
Leptin R1	CCAGGAATGAAGTCCAAACC
ACTB	Tataa Biocenter qA-01-010455
